# Vitamin D Levels Are Associated with Cardiac Autonomic Activity in Healthy Humans

**DOI:** 10.3390/nu5062114

**Published:** 2013-06-10

**Authors:** Michelle C. Mann, Derek V. Exner, Brenda R. Hemmelgarn, Darlene Y. Sola, Tanvir C. Turin, Linda Ellis, Sofia B. Ahmed

**Affiliations:** 1 University of Calgary, 1403 29th St. NW, Calgary, AB T2N 2T9, Canada; E-Mails: mcmann@ucalgary.ca (M.C.M.); brenda.hemmelgarn@albertahealthservices.ca (B.R.H.); 2 Libin Cardiovascular Institute of Alberta, 3330 Hospital Dr. NW, Calgary, AB T2N 2T9, Canada; E-Mails: exner@ucalgary (D.V.E.); dsola@ucalgary.ca (D.Y.S.); turin.chowdhury@ucalgary.ca (T.C.T.); lellis@ucalgary.ca (L.E.)

**Keywords:** vitamin D and cardiovascular disease, vitamin D deficiency, cholecalciferol, cardiac autonomic nervous system, heart rate variability, angiotensin II

## Abstract

Vitamin D deficiency (≤50nmol/L 25-hydroxy vitamin D) is a cardiovascular (CV) risk factor that affects approximately one billion people worldwide, particularly those affected by chronic kidney disease (CKD). Individuals with CKD demonstrate abnormal cardiac autonomic nervous system activity, which has been linked to the significant rates of CV-related mortality in this population. Whether vitamin D deficiency has a direct association with regulation of cardiac autonomic activity has never been explored in humans. *Methods:* Thirty-four (34) healthy, normotensive subjects were studied and categorized based on 25-hydroxy vitamin D deficiency (deficient *vs.* non-deficient, *n =* 7 *vs.* 27), as well as 1,25-dihydroxy vitamin D levels (above *vs.* below 25th percentile, *n =* 8 *vs.* 26). Power spectral analysis of electrocardiogram recordings provided measures of cardiac autonomic activity across low frequency (LF) and high frequency (HF, representative of vagal contribution) bands, representative of the sympathetic and vagal limbs of the autonomic nervous system when transformed to normalized units (nu), respectively, as well as overall cardiosympathovagal balance (LF:HF) during graded angiotensin II (AngII) challenge (3 ng/kg/min × 30 min, 6 ng/kg/min × 30 min). *Results:* At baseline, significant suppression of sympathovagal balance was observed in the 25-hydroxy vitamin D-deficient participants (LF:HF, *p =* 0.02 *vs.* non-deficient), although no other differences were observed throughout AngII challenge. Participants in the lowest 1,25-dihydroxy VD quartile experienced significant withdrawal of inhibitory vagal control, as well as altered overall sympathovagal balance throughout AngII challenge (HF, mean difference = −6.98 ± 3 nu, *p =* 0.05; LF:HF, mean difference = 0.34 ± 0.1, *p =* 0.043 *vs.* above 25th percentile). *Conclusions:* Vitamin D deficiency is associated with suppression of resting cardiac autonomic activity, while low 1,25-dihydroxy vitamin D levels are associated with unfavourable cardiac autonomic activity during an acute AngII stressor, offering a potential pathophysiological mechanism that may be acting to elevate CV risk in in populations with low vitamin D status.

## 1. Introduction

Vitamin D deficiency is influenced by dietary habits, body composition, ethnicity, as well as sun exposure relative to geographical location and altitude, and has been shown to be associated with poor cardiovascular (CV) outcomes and risk [1,2,3]. Vitamin D deficiency has been shown to alter activity of the cardiovascular system and related pressor responses [4] and dysfunction of the cardiac autonomic nervous system (ANS) is predictive of CV risk [5,6,7,8,9,10,11], specifically risk of sudden cardiac death (SCD) [8,10], in humans. These observations suggest a potential link between low vitamin D and unfavourable alterations in cardiac autonomic tone. The cardiac sympathetic and parasympathetic (vagal) limbs of the ANS act in concert with one another to stimulate and inhibit heart rate and contractility, respectively. Though the association between vitamin D and cardiac autonomic tone has not specifically previously been studied in humans, patients with impaired vitamin D synthesis such as those with chronic kidney disease (CKD) demonstrate unfavourable cardiosympathovagal activity characterized by a significant withdrawal of inhibitory vagal activity [5]. Together, these observations suggest a potential link between vitamin D and cardiac autonomic nervous system (ANS) control and therefore CV risk; though whether vitamin D deficiency has a direct influence on cardiac autonomic activity remains unknown in humans. As such, we sought to evaluate the impact of low serum levels of vitamin D metabolites: (a) vitamin D deficiency, defined as <50 nmol/L (or 20 ng/mL: 1 nmol/L = 0.4 ng/mL) 25-hydroxy vitamin D [12]; and (b) low 1,25-dihydroxy vitamin D, defined as within the lowest quartile of 1,25-dihydroxy vitamin D [2] on cardiac autonomic activity in healthy humans.

## 2. Methods

Thirty-four (34) healthy, normotensive volunteer subjects underwent graded angiotensin II (AngII) challenge (3 ng/kg/min × 30 min, 6 ng/kg/min × 30 min). This challenge involved infusion of exogenous AngII, the effector hormone of the renin-angiotensin system (RAS) within the kidney which induces a slight increase in blood pressure (BP) and thus acts as an acute physiological stressor in healthy subjects [13,14]. This study was approved by the Conjoint Health Research Ethics Board (CHREB) at the University of Calgary (project ID #22859) and was conducted in accordance with the principles outlined in the Declaration of Helsinki.

Subjects were recruited year-round via posters on the University of Calgary Health Sciences and Main campuses. Exclusion criteria were hypertension (BP > 140/90 or the use of antihypertensive medications), diabetes (fasting glucose ≥7 mmol/L or the use of hypoglycemic medications), smoking and use of any regular prescription medications. Following collection of verbal and written informed consent, subjects consumed >150 mmol Na^+^/day for three days prior to the study to ensure maximal RAS suppression [15]. All women were studied mid-menstrual cycle, confirmed by counting days from the last menstrual period and measurement of sex hormone levels. All studies were conducted at the University of Calgary Human Physiology Laboratory and commenced at 08:00 with fasting subjects lying supine in a quiet, temperature-controlled room. An 18-gauge peripheral venous cannula was then inserted into each antecubital vein for blood sampling and infusions. BP was monitored throughout the study by an automated sphygmomanometer cuff (GE Healthcare, Dinamap) at 15 min intervals. Following a 90-min equilibration period, subjects underwent graded AngII infusion. 

### 2.1. Cardiac Autonomic Activity

Electrocardiogram (ECG) data were collected during spontaneous, natural breathing with a 3-lead ambulatory Holter monitor (GE Healthcare; Milwaukee, WI, USA) at a sampling frequency of 125 Hz. ECG data was collected continuously throughout the duration of the 180 min study period (baseline × 90 min, 3 ng/kg/min AngII × 30 min, 6 ng/kg/min AngII × 30 min). ECG tracings were screened by a qualified technician for artefacts and irregular QRS complexes in order to exclude any abnormal rhythms. Power spectral density analysis was then calculated to include rhythms with activity within the bandwidth frequency of 0.003–1.7 Hz (MARS v. 7; GE Healthcare; Milwaukee, WI, USA). Absolute values of cardiac autonomic activity (in milliseconds) in the total power (TP), very-low frequency (VLF, 0.003–0.04 Hz), low-frequency (LF, 0.04–0.15 Hz) and high-frequency (HF, 0.15–0.4 Hz) bands were recorded. Both absolute LF and HF parameters were then squared and log-transformed (*ln* ms^2^), as well as converted to normalized units (nu) to account for changes in total autonomic power [16]. Overall cardiosympathovagal balance (LF:HF) was derived automatically within the MARS software by comparing the LF/LF + HF and HF/HF + LF ratios.

While some methodological variation exists within the literature [17], it is widely accepted that power spectral density analysis measurements within the HF frequency bandwidth represents independent contribution from the vagal limb of the cardiac autonomic nervous system [5,16]. While some controversy exists with regards to the remaining frequency domain measures, it has been speculated that activity within the VLF frequency bandwidth may represent contribution influenced by both the RAS [18], and more recently, the vagal limb of the cardiac ANS [19]. Previously, the LF bandwidth has been attributed to the sympathetic limb of the cardiac ANS [16], however this view has been challenged and interpreted as being largely influenced by vagal outflow generated by the baroreceptor reflex [20]. The LF:HF measure is thought to be an overall estimate of the relative balance of autonomic activity within the entire cardiac ANS [16].

### 2.2. Biochemical Analysis

Serum 25-hydroxy vitamin D and parathyroid hormone (PTH) were quantified using chemiluminescence immunoassay techniques (Liaison 25-hydroxyVitamin D Total Assay, Liaison N-TACT PTH, DiaSorin Clinical Assays; Stillwater, MN, USA). Serum 1,25-dihydroxy vitamin D level was determined by a two-step assay involving extraction of vitamin D metabolites followed by competitive radioimmunoassay (1,25-dihydroxy vitamin D 125I RIA kit, DiaSorin Clinical Assays; Stillwater, MN, USA). Plasma renin activity (PRA) was determined by quantification of plasma angiotensin I, the primary product of renin activation, using radioimmunoassay (Plasma Renin Activity 125I, DiaSorin Clinical Assays; Stillwater, MN, USA). AngII plasma levels were measured by standard immunoassay techniques (Quest Diagnostics; San Juan Capistrano, CA, USA). Serum aldosterone levels were determined by radioimmunoassay (Aldosterone Coat a Count Kit, Intermedico; Markham, Ontario, Canada). Serum creatinine and cholesterol were quantified by enzymatic colorimetric assay (Roche/Hitachi Creatinine Plus, CHOD-PAP, HDL-C Plus, and Triglycerides GPO-PAP Kits, Roche Diagnostics; Indianapolis, IN, USA).

### 2.3. Statistical Analysis

Data are expressed as mean ± SE. The primary outcome was the association between 25-hydroxy vitamin D status (deficient *vs.* non-deficient) and cardiac autonomic activity differences at baseline and in response to AngII challenge. The secondary outcome was the association between active 1,25-dihydroxy vitamin D status (below 25th percentile *vs.* above 25th percentile) [2] and changes in cardiac autonomic activity at baseline and in response to AngII challenge. Spearman’s correlational analyses were employed to evaluate the various relationships between relevant variables and outcomes. Differences between the vitamin D groups at specific time points were tested using non-parametric Mann–Whitney U. Linear trends for responses over time were calculated using the non-parametric one-way analysis of variance (Kruskal–Wallis). Within-subject and between-group responses were also analyzed with two-way repeated measures analysis of variance (ANOVA) to account for both the dose of AngII infusion and vitamin D group allocation, with race and gender [21] included as covariates within the model and multiple comparison corrections used as appropriate. All test assumptions were tested and verified before conducting analyses utilizing SPSS (IBM; v. 19) with a significance level of α ≤ 0.05.

## 3. Results

### 3.1. Subject Characteristics

Subject characteristics are described in Table 1. All subjects were normotensive irrespective of 25-hydroxy or 1,25-dihydroxy vitamin D serum concentration, though vitamin D deficient subjects demonstrated greater parathyroid hormone levels (*p =* 0.046) and lower epinephrine levels (*p =* 0.019). There was a trend towards lower self-reported Caucasian race and lower 1,25-dihydroxy vitamin D levels in the Vitamin D deficient group but this did not achieve statistical significance. When subjects were stratified by 1,25-dihydroxy vitamin D status, similar findings were observed.. No association between 25-hydroxy vitamin D and 1,25-dihydroxy vitamin D was observed (*r* = 0.061, *p =* 0.73). 

**Table 1:** Baseline characteristics.

		Stratification by 25-hydroxy vitamin D status			Stratification by 1,25-dihydroxy vitamin D status		
	All Subjects (*n =* 34)	Deficient <50 nmol/L (*n =* 7)	Non-Deficient >50 nmol/L (*n =* 27)	*p*-value	Below 25th percentile <76 pmol/L (*n =* 8)	Above 25th percentile >76 pmol/L (*n =* 26)	*p*-value
Age (years)	38 ± 2	37 ± 5	38 ± 3	0.96	37 ± 4	38 ± 3	0.92
Caucasian (%)	25 (74%)	4 (57%)	21 (77%)	0.08	7 (88%)	18 (69%)	0.56
Female (%)	22 (65%)	5 (71%)	17 (63%)	0.69	6 (75%)	16 (62%)	0.22
BMI (kg/m^2^)	26 ± 1	27 ± 1	25 ± 1	0.43	27 ± 2	25 ± 1	0.21
SBP (mmHg)	115 ± 2	112 ± 4	115 ± 3	0.63	115 ± 2	115 ± 3	0.37
DBP (mmHg)	68 ± 1	64 ± 5	69 ± 2	0.28	70 ± 3	68 ± 2	0.44
MAP (mmHg)	84 ± 2	80 ± 4	84 ± 2	0.37	85 ± 2	83 ± 2	0.41
25-hydroxy vitamin D (nmol/L)	71 ± 4	38 ± 2	81 ± 4	<0.001	61 ± 7	74 ± 5	0.16
1,25-dihydroxy vitamin D (pmol/L)	105 ± 6	86 ± 9	107 ± 7	0.15	64 ± 3	117 ± 6	<0.001
PTH (ng/L)	37 ± 2	45 ± 9	34 ± 2	0.046	33 ± 6	38 ± 3	0.29
Serum calcium (mmol/L)	2.2 ± 0.01	2.3 ± 0.04	2.2 ± 0.01	0.47	2.2 ± 0.03	2.2 ± 0.02	0.49
Serum phosphate (mmol/L)	1.0 ± 0.03	0.9 ± 0.08	1.01 ± 0.03	0.35	1.03 ± 0.04	0.97 ± 0.04	0.29
HDL (mmol/L)	1.4 ± 0.05	1.3 ± 0.1	1.5 ± 0.05	0.21	1.45 ± 0.07	1.4 ± 0.06	0.62
LDL (mmol/L)	2.2 ± 0.1	1.8 ± 0.2	2.3 ± 0.2	0.17	2.1 ± 0.4	2.2 ± 0.2	0.65
NE (nmol/L)	1.3 ± 0.2	1.2 ± 0.06	1.3 ± 0.2	0.79	1.5 ± 0.3	1.3 ± 0.2	0.37
Epi (pmol/L)	97 ± 13	69 ± 11	117 ± 17	0.019	76 ± 13	103 ± 15	0.64
Urinary sodium (mmol/day)	373 ± 20	355 ± 48	379 ± 25	0.65	348 ± 25	380 ± 27	0.40
Serum creatinine (umol/L)	69 ± 4	69 ± 7	70 ± 3	0.92	70 ± 5	67 ± 4	0.98
PRA (ng/L/s)	0.21 ± 0.03	0.23 ± 0.04	0.20 ± 0.03	0.48	0.15 ± 0.03	0.23 ± 0.03	0.19
Ang II (ng/L)	18 ± 1	18 ± 3	16 ± 1	0.38	18 ± 2	18 ± 1	0.81
Aldo (pmol/L)	106 ± 10	90 ± 8	110 ± 12	0.61	99 ± 14	109 ± 12	0.91

BMI, body mass index; SBP, systolic blood pressure; DBP, diastolic blood pressure; MAP, mean arterial pressure; PTH, parathyroid hormone; HDL, high-density lipoprotein (cholesterol); LDL, low-density lipoprotein (cholesterol); NE, norepinephrine; Epi, epinephrine; PRA, plasma renin activity Ang II, angiotensin II; Aldo, aldosterone. All data are expressed as mean ± SE unless otherwise indicated.

### 3.2. Cardiac Autonomic Responses in 25-Hydroxy Vitamin D Deficient vs. Non-Vitamin D Deficient Subjects

**Figure 1:**
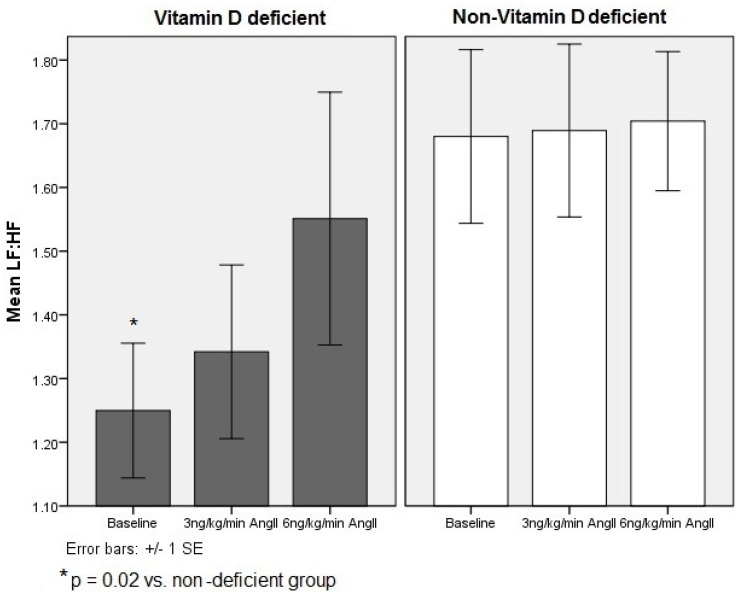
Cardiosympathovagal modulation during angiotensin II challenge, by vitamin D status LF:HF, low- to high-frequency ratio representative of cardiosympathovagal balance.

At baseline, significant suppression of overall resting sympathovagal balance was observed in the 25-hydroxy vitamin D deficient participants (LF:HF, *p =* 0.02) (Figure 1). During AngII infusion, no significant differences between groups at a specific time point were noted. Further, no significant trends in responses over time were observed within either group (Table 2). Two-way repeated measures ANOVA revealed no significant within-subject or between-group contrasts despite controlling for potential confounders, suggesting that differences in 25-hydroxy vitamin D did not alter cardiac autonomic responses to AngII.

**Table 2:** Cardiac autonomic response to angiotensin II challenge by 25-hydroxy vitamin D status.

	Baseline	3 ng/kg/min AngII	6 ng/kg/min AngII
**Heart rate (bpm)**			
*Vitamin D deficient Non-vitamin D deficient*	62 ± 3 57 ± 2	56 ± 3 56 ± 3	65 ± 4 58 ± 2
**TP (ms)**			
*Vitamin D deficient*	56 ± 7	66 ± 7	63 ± 8
*Non-vitamin D deficient*	69 ± 6	74 ± 7	73 ± 7
**VLF (ms)**			
*Vitamin D deficient*	39 ± 6	48 ± 6	47 ± 6
*Non-vitamin D deficient*	51 ± 5	55 ± 7	56 ± 5
**LF (ms)**			
*Vitamin D deficient*	31 ± 4	34 ± 4	33 ± 5
*Non-vitamin D deficient*	37 ± 4	37 ± 3	37 ± 3
**LF (*ln* ms^2^)**			
*Vitamin D deficient*	6.76 ± 0.3	6.93 ± 0.3	6.84 ± 0.3
*Non-vitamin D deficient*	6.99 ± 0.2	6.97 ± 0.2	7.00 ± 0.2
**LF (nu)**			
*Vitamin D deficient*	61 ± 5	58 ± 3	62 ± 4
*Non-vitamin D deficient*	67 ± 3	66 ± 3	68 ± 2
**HF (ms)**			
*Vitamin D deficient*	25 ± 3	26 ± 3	24 ± 4
*Non-vitamin D deficient*	25 ± 3	24 ± 3	23 ± 2
**HF (*ln* ms^2^)**			
*Vitamin D deficient*	6.36 ± 0.3	6.40 ± 0.3	6.06 ± 0.6
*Non-vitamin D deficient*	6.12 ± 0.2	6.02 ± 0.2	5.98 ± 0.2
**HF (nu)**			
*Vitamin D deficient*	41 ± 7	37 ± 4	32 ± 5
*Non-vitamin D deficient*	33 ± 3	28 ± 3	26 ± 2
**LF:HF **			
*Vitamin D deficient*	1.25 ± 0.1	1.34 ± 0.2	1.55 ± 0.2
*Non-vitamin D deficient*	1.66 ± 0.1 ^†^	1.72 ± 0.1	1.74 ± 0.1

TP, total power; VLF, very-low frequency; LF, low-frequency; HF, high-frequency. All data are expressed as mean ± SE unless otherwise indicated. Vitamin D deficient (*n =* 7), non-vitamin D deficient (*n =* 27). * *p* < 0.05 *vs.* response from baseline; ^†^ *p* < 0.05 *vs.* vitamin D deficient value at this time point.

### 3.3. Cardiac Autonomic Responses below vs. above 25th Percentile 1,25-Dihydroxy Vitamin D Subjects

**Figure 2:**
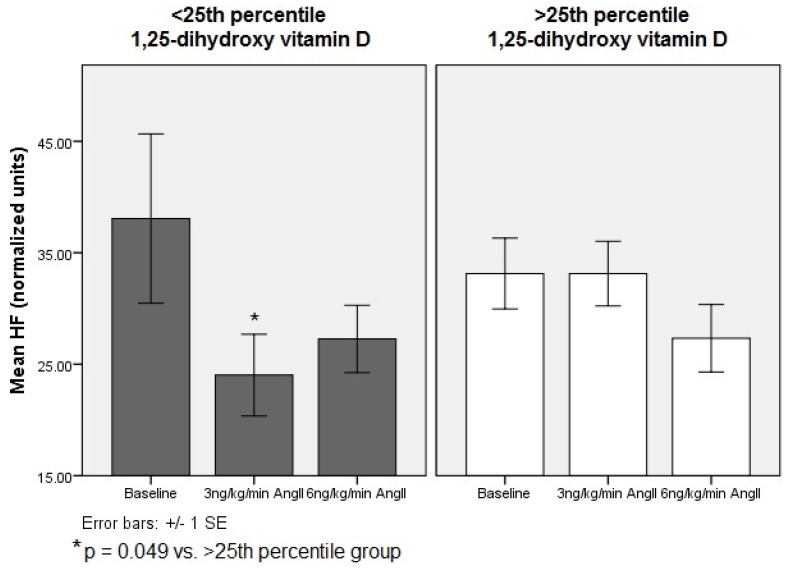
Comparison of cardiac vagal autonomic activity between 1,25-dihydroxy vitamin D groups during angiotensin II challenge HF, high-frequency representative of cardiac parasympathetic activity; nu, normalized units.

At baseline, there were no significant differences in cardiac autonomic activity between groups. In response to 30 min of AngII challenge, subjects with 1,25-dihydroxy vitamin D levels below the 25th percentile demonstrated loss of vagal tone as demonstrated by a decrease in HF (nu) compared to subjects with 1,25-dihydroxy vitamin D levels above the 25th percentile (*p =* 0.05). As a consequence of the loss of vagal tone in response to the stressor, subjects with 1,25-dihydroxy vitamin D levels below the 25th percentile were unable to maintain overall sympathovagal balance compared to those with 1,25-dihydroxy vitamin D levels above the 25th percentile (*p =* 0.043) (Table 3). Comparison of the higher and lower 1,25-dihydroxy vitamin D groups revealed that 1,25-hydroxy vitamin D status was associated with differences in the LF, HF, and LF:HF responses across AngII doses, specifically at the 3 ng/kg/min dose (LF nu, mean difference = 8.84 ± 4 nu, *p =* 0.034; HF nu, mean difference = −6.98 ± 3 nu, *p =* 0.049 (Figure 2); LF:HF, mean difference = 0.34 ± 0.1, *p =* 0.080).

**Table 3:** Cardiac autonomic response to angiotensin II challenge by 1,25-dihydroxy vitamin D status.

	Baseline	3 ng/kg/min AngII	6 ng/kg/min AngII
**Heart rate (bpm)**	59 ± 3 58 ± 2	60 ± 2 56 ± 2	62 ± 3 58 ± 2
*Below 25th percentile*			
*Above 25th percentile*			
**TP (ms)**	70 ± 15 66 ± 5	83 ± 16 69 ± 6	85 ± 18 67 ± 5
*Below 25th percentile*			
*Above 25th percentile*			
**VLF (ms)**	50 ± 12 48 ± 4	66 ± 13 50 ± 5	58 ± 11 53 ± 4
*Below 25th percentile*			
*Above 25th percentile*			
**LF (ms)**	40 ± 8 35 ± 3	43 ± 8 34 ± 3	40 ± 10 35 ± 2
*Below 25th percentile*			
*Above 25th percentile*			
**LF (*ln* ms^2^)**	7.04 ± 0.4 6.91 ± 0.2	7.28 ± 0.4 6.87 ± 0.2	6.89 ± 0.5 6.99 ± 0.2
*Below 25th percentile*			
*Above 25th percentile*			
**LF (nu)**	67 ± 4 65 ± 3	72 ± 4 62 ± 3	69 ± 4 66 ± 2
*Below 25th percentile*			
*Above 25th percentile*			
**HF (ms)**	25 ± 5 25 ± 3	23 ± 4 25 ± 2	21 ± 5 24 ± 2
*Below 25th percentile*			
*Above 25th percentile*			
**HF (*ln* ms^2^)**	6.15 ± 0.4 6.17 ± 0.2	6.04 ± 0.3 6.12 ± 0.2	5.79 ± 0.4 6.06 ± 0.2
*Below 25th percentile*			
*Above 25th percentile*			
**HF (nu)**	37 ± 7 34 ± 3	23 ± 3 32 ± 3 ^†^	25 ± 3 28 ± 3
*Below 25th percentile*			
*Above 25th percentile*			
**LF:HF **	1.6 ± 0.1 1.6 ± 0.1	1.9 ± 0.2 1.6 ± 0.1 ^†^	1.8 ± 0.2 0.7 ± 0.1
*Below 25th percentile*			
*Above 25th percentile*			

TP, total power; VLF, very-low frequency; LF, low-frequency; HF, high-frequency. All data are expressed as mean ± SE unless otherwise indicated. Below 25th percentile (*n =* 8), above 25th percentile (*n =* 26). * *p* < 0.05 *vs.* response from baseline; ^†^ *p* < 0.05 *vs.* below 25th percentile at this time point.

### 3.4. Systemic Responses to Angiotensin II Challenge

Throughout the AngII challenge, BP parameters increased similarly in all subjects regardless of vitamin D stratification. Further, PRA decreased from baseline while aldosterone levels increased from baseline (Table 1) as expected throughout the AngII challenge, with no significant differences in PRA or aldosterone activity between either vitamin D stratification groups. No significant trends or repeated measures comparisons were observed in any BP or RAS parameter.

## 4. Discussion

This is the first study to examine the relationship between 25-hydroxy vitamin D, the barometer of vitamin D status, as well as 1,25-dihydroxy vitamin D, the activated form of vitamin D, with cardiac autonomic activity at baseline and in response to a vascular stressor in humans. Our key findings were: (1) vitamin D deficiency as determined by 25-hydroxy vitamin D levels in healthy humans were associated only with a suppression of resting overall cardiosympathovagal balance; and (2) lower levels of 1,25-dihydroxy vitamin D, the activated form of vitamin D, were associated with unfavourable shifts in cardiosympathovagal balance, driven by exaggerated withdrawal of cardioprotective vagal tone, in response to AngII challenge.

### 4.1. Vitamin D and Cardiovascular Risk

Vitamin D deficiency (<50 nmol/L 25-hydroxy vitamin D) has been highlighted in numerous studies involving high-risk CV disease populations [22,23], suggesting that low vitamin D status may be a potentially significant and treatable risk factor. In an observational study by Wang and colleagues [24], normotensive subjects with 25-hydroxy vitamin D serum concentrations of <37.5 nmol/L demonstrated a 62% increase in CV disease risk over a 5.4-year follow-up compared to those with higher levels. Similarly, Dobnig *et al.* reported that in 3258 patients referred for coronary angiography, those within the lowest quartiles of 25-hydroxy (<35 nmol/L) or 1,25-dihydroxy vitamin D (<51 pmol/L) were at highest risk of sudden cardiac death [2], hence the rationale to also stratify subjects by the lowest 1,25-dihydroxy vitamin D quartile in the present study. Moreover, recent studies of healthy adults have shown that lower 25-hydroxy vitamin D were independently associated with an increased risk of SCD [3,24], suggesting that vitamin D status is important even in populations without established pathologies. 

### 4.2. Vitamin D and Cardiac Autonomic Activity

Our results suggest that low serum levels of vitamin D may be associated with a decline in cardioprotective vagal tone in response to an acute vascular stressor, largely through the action of the active 1,25-dihydroxy vitamin D metabolite. Active 1,25-dihydroxy vitamin D has been identified in some mammalian studies as having the potential to influence cardiac autonomic activity [25,26,27]. These studies demonstrate that cardiac myocytes isolated from vitamin D receptor knockout mice display accelerated rates of contraction as compared with wild type. Further, exposure to 1,25-dihydroxy vitamin D directly attenuated this rapid contractility in the wild-type but not the knockout cardiac myocyte [27], suggesting a relationship between 1,25-dihydroxy vitamin D and vagal inhibitory outflow. Moreover, activated vitamin D is thought to have the ability to diffuse across the blood–brain barrier, implicating a role for 1,25-dihydroxy vitamin D in augmenting autonomic vagal control by binding directly to nuclear vitamin D receptors in the adrenergic neurons located centrally in the spinal cord and brain tissue [26]. 

In support of our novel observations in healthy humans, patients with chronic kidney disease (CKD) demonstrate chronic RAS upregulation [28,29], as well as altered cardiac autonomic activity defined primarily by extreme vagal insufficiency [5]. This same population has a diminished capacity to convert 25-hydroxy vitamin D to 1,25-dihydroxy vitamin D due to decreased renal 1-α hydroxylase capacity [1]; however, CV mortality in this population declines following treatment with exogenous 1,25-dihydroxy vitamin D supplementation [30]. Moreover, a recent study by Kendrick *et al.* demonstrates that both CV death and time to initiation of dialysis in individuals with advanced stages of CKD are independently associated with serum 1,25-dihydroxy vitamin D levels. The authors did not find these associations when stratifying subjects by 25-hydroxy vitamin D status [31]. As such, the withdrawal of vagal tone observed in low 1,25-dihydroxy vitamin D subjects during AngII challenge may mimic the loss of vagal tone that has been noted in the end-stage renal disease population [5]. These patients are characterized by severe vitamin D deficiency [1]—particularly low serum concentrations of 1,25-dihydroxy vitamin D due to compromised 1-alpha hydroxylase activity housed within the functional renal tissue—as well as chronically upregulated RAS activity. 

Further, dysfunction of cardiac autonomic tone, specifically vagal tone, has been shown to be predictive of sudden cardiac death [5,8,9,10]. Wolf and colleagues have demonstrated a survival advantage and specifically a decrease in cardiovascular death in hemodialysis patients taking 1,25-dihydroxy vitamin D supplementation irrespective of 25-hydroxy vitamin D level [30]. These observations support our findings which suggest that the activated 1,25-dihydroxy vitamin D metabolite may be capable of altering cardiac autonomic tone in individuals experiencing upregulation of the RAS and that low 1,25-dihydroxy vitamin D levels contribute to the loss of vagal outflow, and therefore high CV risk, observed in these populations.

### 4.3. Challenges and Limitations

In humans, vitamin D is part of a complex mineral metabolism involving additional measures that have been shown to influence CV risk, including PTH, phosphate, and calcium [32]. While PTH levels were significantly elevated in the 25-hydroxy vitamin D deficient group, PTH and other mineral metabolism variables were within narrow, healthy ranges. Further, our study was performed over different seasons, though the season of study was not found to be correlated to vitamin D levels. Salt intake has been shown to influence cardiac ANS activity [33]. However, all subjects were in high-salt balance to ensure a maximal suppression of basal RAS levels, allowing for meaningful comparisons between subjects and direct observation of the impact of exogenous AngII infusion on cardiac autonomic activity. The study sample size was limited and only included non-smoking healthy subjects who were non-obese, normotensive, non-diabetic with normal kidney function, limiting the generalizability of our study results to the general population. However, by studying a healthier population, we aimed to examine the impact of various vitamin D metabolites on cardiac autonomic tone at baseline and in response to AngII while minimizing confounding factors. Furthermore, this group reflects the population referenced by the Endocrine Society which defines vitamin D deficiency as <50 nmol/L 25-hydroxy vitamin D [12]. We found that allocation to the 25-hydroxy vitamin D deficiency group was not correlated with allocation to the low 1,25-dihydroxy vitamin D group, a finding that is consistent with previous reports, thereby suggesting that the potential influences of individual dietary calcium consumption, PTH levels, or individual 1-α hydroxylase capacity may play a role in determining an individual’s 25-hydroxy and 1,25-dihydroxy vitamin D levels [1,32]. Next, our results appeared most significant in response to the first graded dose of AngII, perhaps representing a threshold effect that persisted through the second dose. Lastly, while we cannot comment on the role of vitamin D supplementation in improving cardiac responses to AngII challenge or other stressors, our study adds to the growing body of literature supporting a link between vitamin D metabolite levels and overall CV outcomes.

### 4.4. Implications

To our knowledge, there have been no previous studies investigating the potential link between vitamin D metabolites and modulation of the cardiac ANS, a risk factor for poor CV outcomes in healthy and diseased populations [2,3,4,7,8,9,10,11,16]. Our study illustrates a unique relationship between low 25-hydroxy vitamin D levels and depressed baseline cardiac autonomic activity, as well as low 1,25-dihydroxy vitamin D and unfavourable cardiosympathovagal shifts during acute AngII challenge, which allows for unique insight into a pathophysiological mechanism that may be acting to elevate CV risk, particularly in renal populations with chronic upregulation of the RAS, in addition to impaired 1-α hydroxylase activity [22,23,24,25,26,27,28,29,30,31]. While larger studies involving supplementation are required, dysregulation of vitamin D metabolism remains a potentially treatable condition that warrants further investigation in high-risk populations.
